# ‘Covering the tail’ after stopping efavirenz-based antiretroviral therapy

**DOI:** 10.4102/sajhivmed.v21i1.1036

**Published:** 2020-01-27

**Authors:** Gary Maartens

**Affiliations:** 1 Division of Clinical Pharmacology, Department of Medicine, University of Cape Town, Cape Town, South Africa

Single-dose nevirapine for the prevention of mother-to-child transmission (PMTCT) was associated with the development of non-nucleoside reverse transcriptase inhibitor (NNRTI) resistance mutations in a high proportion of women because of nevirapine’s low genetic barrier to resistance. The hypothesis that dual nucleoside reverse transcriptase inhibitors (NRTIs) given for a short period to ‘cover the tail’ of slowly declining nevirapine concentrations would reduce the risk of emergent NNRTI resistance mutations was borne out by two randomised controlled trials of a single dose of tenofovir plus emtricitabine and 4–7 days of zidovudine plus lamivudine.^1,2^

The practice of ‘covering the tail’ then migrated to stopping NNRTI-based combination antiretroviral therapy (ART) started during pregnancy for PMTCT, which was ‘option B’ in World Health Organization (WHO) guidelines for women who did not qualify for long term ART based on CD4 count thresholds. ‘Covering the tail’ was also recommended in guidelines, for all patients stopping NNRTI-based ART.

Is there evidence that ‘covering the tail’ after stopping NNRTI-based ART reduces the risk of developing NNRTI resistance, and is the practice relevant in the era of ART for all?

In this edition of the journal, Ajibola et al.^3^ suggest that ‘covering the tail’ after stopping efavirenz-based ART might reduce the risk of developing NNRTI resistance (please see https://doi.org/10.4102/sajhivmed.v21i1.1023). They conducted a retrospective study of women stopping efavirenz-based ART started in pregnancy as ‘option B’ in Botswana and found that women who received a week of tenofovir plus emtricitabine after stopping ART had less NNRTI resistance. However, their study findings just failed to achieve statistical significance, likely because of the small sample size. Another problem with the study is that allocation to ‘covering the tail’ was not random. Because of these study limitations, we still lack good evidence that that ‘covering the tail’ after stopping NNRTI-based ART reduces the risk of developing NNRTI resistance.

The rationale for ‘covering the tail’ after stopping NNRTI-based ART is that efavirenz and nevirapine have longer half-lives than the older NNRTIs. However, data from a pharmacokinetic study question the need for ‘covering the tail’ with a regimen of tenofovir, emtricitabine and efavirenz: the half-lives of intracellular tenofovir-diphosphate and emtricitabine-triphosphate (the active drugs) is 164 and 39 h, respectively, compared with 92 h for plasma efavirenz.^4^ The half-life of efavirenz is variable, largely explained by polymorphisms in *CYP2B6*, which encodes the main metabolising enzyme: the prevalence of the *CYP2B6* slow metaboliser genotype, which results in a longer efavirenz half-life, is about 20% in South Africa.^5^ Therefore, in order to rationally ‘cover the tail’ after stopping efavirenz-based ART, the *CYP2B6* metaboliser genotype should be known and an additional dose or two of emtricitabine should be given; however, emtricitabine is only available in the region co-formulated with tenofovir and genotype data are almost never known. There is no good pharmacokinetic rationale for ‘covering the tail’ with a week of tenofovir plus emtricitabine, which was recommended in the Botswana paper. Furthermore, the time to viral rebound after stopping ART in patients who have achieved virologic suppression is variable, but typically takes weeks rather than days^6^ and is longer with NNRTI-based ART.^7^ Finally, there is rarely a medical indication to interrupt ART in the current era of ART for all. For these reasons, the SA HIV Clinicians Society guidelines on ART no longer recommend ‘covering the tail’ if ART is interrupted.

## References

[1] Chi BH, Sinkala M, Mbewe F, et al Single-dose tenofovir and emtricitabine for reduction of viral resistance to non-nucleoside reverse transcriptase inhibitor drugs in women given intrapartum nevirapine for perinatal HIV prevention: An open-label randomised trial. Lancet. 2007;370(9600):1698–1705. 10.1016/S0140-6736(07)61605-5 17997151

[2] McIntyre JA, Hopley M, Moodley D, et al Efficacy of short-course AZT plus 3TC to reduce nevirapine resistance in the prevention of mother-to-child HIV transmission: A randomized clinical trial. PLoS Med. 2009;6(10):e1000172 10.1371/journal.pmed.1000172 19859531 PMC2760761

[3] Ajibola G, Maruapula D, Rowley C, et al ‘Drug resistance after cessation of efavirenz-based antiretroviral treatment started in pregnancy. S Afr J HIV Med. 2020;21(1), a1023 10.4102/sajhivmed.v21i1.1023 PMC7059240 32158555

[4] Jackson A, Moyle G, Watson V, et al Tenofovir, emtricitabine intracellular and plasma, and efavirenz plasma concentration decay following drug intake cessation: Implications for HIV treatment and prevention. J Acquir Immune Defic Syndr. 2013;62(3):275–281. 10.1097/QAI.0b013e3182829bd0 23274933

[5] Sinxadi PZ, Leger PD, McIlleron H, et al Pharmacogenetics of plasma efavirenz exposure in HIV-infected adults and children in South Africa. Br J Clin Pharmacol. 2015;80(1):146–156. 10.1111/bcp.12590 25611810 PMC4500334

[6] Treasure GC, Aga E, Bosch RJ, et al Brief report: Relationship among viral load outcomes in HIV treatment interruption trials. J Acquir Immune Defic Syndr. 2016;72(3):310–313. 10.1097/QAI.0000000000000964 26910502 PMC4911279

[7] Li JZ, Etemad B, Ahmed H, et al The size of the expressed HIV reservoir predicts timing of viral rebound after treatment interruption. AIDS. 2016;30(3):343–353. 10.1097/01.aids.0000499516.66930.89 26588174 PMC4840470

